# Utilization of Melt Fracture Phenomenon for the Preparation of Shark Skin Structured Hydrophobic Film

**DOI:** 10.3390/polym13244299

**Published:** 2021-12-09

**Authors:** Bin Tang, Yaoyu Yue, Zipeng Gai, Yao Huang, Ying Liu, Xiaolong Gao, Jingyao Sun, Daming Wu

**Affiliations:** 1 College of Mechanical and Electrical Engineering, Beijing University of Chemical Technology, Beijing 100029, China; 2019200596@mail.buct.edu.cn (B.T.); 2019210406@mail.buct.edu.cn (Y.Y.); 2019200672@mail.buct.edu.cn (Z.G.); huangyao@mail.buct.edu.cn (Y.H.); liuying@mail.buct.edu.cn (Y.L.); 2 State Key Laboratory of Organic-Inorganic Composites, Beijing University of Chemical Technology, Beijing 100029, China

**Keywords:** melt fracture, hydrophobic, biomimetic, shark skin structure

## Abstract

With the application of biomimetic shark skin microstructures with hydrophobicity in microfluidics, sensors and self-cleaning materials, microstructure processing methods are increasing. The preparation process has higher requirements for processing cost and efficiency. In this paper, linear low-density polyethylene (LLDPE) hydrophobic films were prepared with the help of melt fracture phenomenon. The equipment is a self-made single screw extruder. By adjusting the process parameters, the biomimetic shark skin structured LLDPE films with good hydrophobic property can be obtained. The surface microstructure shape of the product is related to kinds of additive, die temperature and screw speed. When AC5 was selected as an additive, the optimal processing parameter was found to be 160 °C die temperature and 80 r/min screw speed. A contact angle of 133° was obtained in this situation. In addition, the influences of die temperature and screw speed on the size of shark skin structure were also systematically investigated in this paper. It was found that the microstructure surface with hierarchical roughness had a better hydrophobic property.

## 1. Introduction

The surface of the material with a particular shape of microstructure characteristics has some specific physical and chemical functions. The microstructure characteristics has the property of hydrophobic/hydrophilic, drag reduction, stealth, light diffusion and so on. It has broad application prospects in aviation, microfluidic chips, optical components, water and oil separation, self-cleaning materials, and sensors [1,2,3,4,5,6,7,8,9,10]. At present, the research based on microstructure manufacturing mainly adopts two structures: groove structure and biological simulation shield scale structure [11,12,13,14,15,16]. Microfabrication technology is the main technical means of groove structure preparation. It includes LIGA lithography technology, micro-EDM, diamond processing, and femtosecond laser micro-nano processing technology [17,18,19]. The processing of the biological simulation shield scale structure mainly relies on direct copy manufacturing. Its preparation methods mainly include micro-imprinting, micro-plastic casting, micro-electroforming, and soft engraving technology [20,21,22,23,24,25,26,27,28]. Comprehensive research on functional surfaces of polymer microstructures at home and abroad, it shows that the key to the preparation of biomimetic microstructures lies in the precise shaping of the structure. Furthermore, the microstructure has some weather resistance and mechanical strength. The challenge is in the controllability and repeatability of the microstructure’s feature size. In addition, the efficiency and cost of processing must be examined in light of the industry’s needs. Although the following two microstructure preparation methods are well-established and capable of processing a wide range of complex three-dimensional microstructures, and they still have shortcomings in terms of processing cost and efficiency. Therefore, it is necessary to explore a high-efficiency microstructure processing method.

Most polymer products are prepared by extrusion molding. When the polymer reaches a certain critical state during the extrusion flow process, various flow instabilities often appear. In the past decades, in the study of polymer extrusion processing, it has been found that some densely entangled polymers, such as polyethylene, have the defect of flow instability during the extrusion process. This will lead to a melt fracture phenomenon [29,30,31]. Among these, the most prominent are some linear polymers such as LLDPE [32] and HDPE [33]. With the increase of shear rate, it usually shows three different forms of distortion. When the shear rate is low, the surface of the extrudate is smooth. As the shear rate increases, the surface of the extrudate begins to periodically show some small-amplitude and high-frequency corrugated disturbances. This phenomenon is commonly referred to as “shark skin distortion”. A further rise in the shear rate will result in melt wall slippage and a rapid increase in the melt flow rate. Rough parts and smooth areas alternate on the extrudate morphology. This is referred to as a “stick-slip transition”. Finally, when the shear rate is high enough, the extrudate has an overall irregular distortion, which is also called “overall distortion”.

The mechanism of melt extrusion distortion has not yet been unified. Wu et al. [34,35,36,37] studied the concentration effect of melt stress. He believed that the stress concentration effect in the capillary inlet area was the main reason for the overall distortion and even rupture of the melt. Weill [38] found that the occurrence of shark skin distortion was due to the periodic storage and release of melt surface tension, resulting in different speeds and degrees of stress recovery on the surface and inside the polymer. Wang [39] further explored the periodic variation in the stress on the melt at the exit of the capillary. They observed the effect of this change on the stick-slip transition of the polymer. It is believed that when the stress of the melt at the exit of the capillary was less than a certain critical value, the polymer molecular chain slipped relative to the capillary wall. This led to the occurrence of a wall slip phenomenon. When the adsorption chain adheres to the pipe wall again, the outlet stress increases, which makes the melt adhere to the pipe wall again. This led to the occurrence of stick-slip transition.

**Figure 1:**
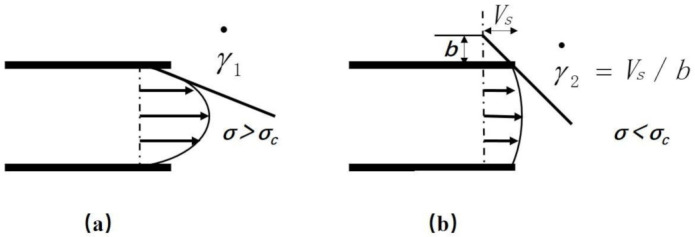
The interface state of fluid dynamics when the polymer shear rate changes at the die exit. (**a**) Viscous state, (**b**) Slip state.

The “shark skin distortion” has self-similarity. The average wavelength (distance between adjacent ribs) and the average depth of the ribs present a linear relationship, and there is a linear relationship between the wavelength and the relaxation time. When the polymer shear rate at the die exit between γ_1_ and γ_2_, the fluid dynamic interface conditions oscillate between the viscous state (Figure 1a) and the sliding state (Figure 1b), which can produce pressure fluctuations. When the polymer/wall interface is in a viscous state (a), the wall stress σ increases. When the wall stress exceeds the critical value σ_c_ of the interface stick-slip transition, it will change from the viscous state (a) to the slip state (b). When the wall slides, the wall stress σ begins to decrease. When σ < σ_c_, the stress near the outlet drops. The polymer molecules are adsorbed and connected to the flow chain to resume entanglement. The polymer/wall interface returns to a viscous state (a). Constant changes between the viscous state (a) and the slip state (b) causes the instability of the melt near the exit of the extrusion die. When the polymer surface is extruded, a certain regular arrangement of microstructures is produced. The size and arrangement of this microstructure are related to wall stress and material parameters.

Studies have shown that the microstructure of “shark skin” can impart extremely low adhesion to the surface of the material, so that the material has ultra-high hydrophobic and oleophobic properties. Frohnapfel [40] simplified the microgrooves on the shark skin surface and performed numerical simulations, which proved that the turbulent dissipation rate between the grooves had an important influence on the drag reduction effect. Zhang [41] used hot embossing to replicate the outer surface of shark skin in a large area. The water cylinder resistance experiment proved that the maximum drag reduction rate of the bionic shark skin could reach 8.25%. Lin [42] replicated the bionic shark skin structure on the outer layer of the PDMS membrane, which not only had super hydrophobicity, but also inhibited bacterial adhesion and accelerated wound healing. By this means, we can create and precisely control the process conditions that produce similar “shark skin” microstructures. In this way, a microstructures with a certain arrangement pattern can be formed on the surface of the polymer, especially on the inner wall of the pipe. By using the shark skin distortion that needs to be avoided in precision extrusion molding, stable microstructure molding on the polymer surface is realized. Therefore, we must find out the process parameters of shark skin distortion and the relationship between shark skin distortion and hydrophobic angle.

The effects of varied proportions of different additives, the temperature of the extrusion die, and the screw speed on shark skin distortion were investigated in this article, utilizing polyethylene as the material. Through a comparison of the material surface contact angle and the size and shape of the surface microstructure, the optimal hydrophobic structure was found, and the optimal processing parameters were obtained. This has a very important guiding significance for subsequent continuous processing, inner wall hydrophobic pipes and other specific applications. At the same time, it is also convenient for the follow-up to further explore the mechanism of shark skin distortion.

## 2. Experimental Section

### 2.1. Materials

In the experiment, LLDPE 7042 (Maoming Petrochemical, Maoming, China) was used as the base material, and different additives were selected and blended. The melt flow rate of LLDPE is 2 g/10 min and the density is 0.92 g/cm^3^. It has a high transparency and strength. It contains a small amount of anti-blocking agent and smoothing agent, and has good extrusion performance. Its film appearance grade and cleanliness also have good performance. Additives include AC3 (erucamide antiblocking agent, produced from Yisheng Chemical, Dongguan, China), AC5 (oleamide antiblocking agent, produced from Yisheng Chemical, Dongguan, China), AC15 (silicon dioxide and erucamide antiblocking agent, produced from Yisheng Chemical, Dongguan, China), and 180 (polyethylene antiblocking agent, produced from Dinghai Plastic Chemical, Dongguan, China).

### 2.2. Preparation of the Hydrophobic Surface of Shark Skin

**Figure 2:**
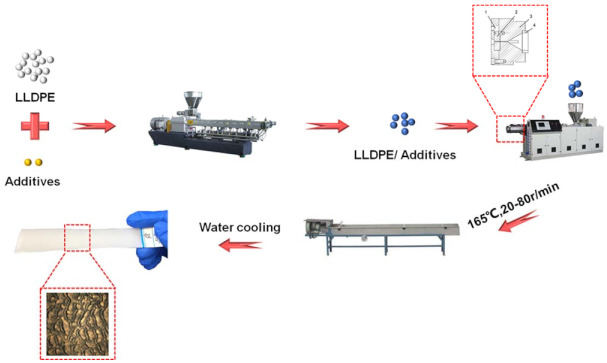
Preparation process of shark skin structured hydrophobic film.

The experiment was mainly divided into six steps: blending granulation, designing extruder head, heating up, increasing speed and sampling, changing additives and sampling, changing temperature and sampling. The preparation process is shown in Figure 2.

Step 1. LLDPE and additives were blended and pelletized through a twin-screw extruder (ZSK-25, Coperion, Shanghai, China). The blending ratio was LLDPE 7042/additive (90/10). The control temperature of each section was: 170 °C, 175 °C, 180 °C, 185 °C, 190 °C, 190 °C, 190 °C, 190 °C, 190 °C, 190 °C.

**Figure 3:**
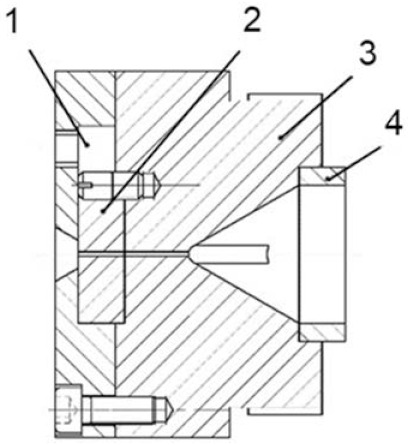
Schematic diagram of the extruder head: 1. Die gland; 2. Detachable metal; 3. Machine head body; 4. Perforated plate.

Step 2. The design of the single screw extruder head. The single-screw extruder used in the experiment is self-developed. Taking into account the influence of the size of the extrusion die on the distortion of shark skin, a set of thin die runners were designed, as shown in Figure 3. The head of the modified extruder was mainly divided into four parts: die gland, detachable metal, machine head body and perforated plate. Among them, the die gland was mainly used to fix the detachable metal, which plays a role of sealing. It was worth mentioning that a part of the space was left in the upper part of the die gland and the upper end of the detachable metal. This area had a good effect when the extrusion flow was intervened by external mechanical means. The detachable metal part can be made of general metal materials, a special gas-permeable metal, or metal treated on the inner wall. Because the flow channel here was the key location for melt fracture and stick-slip transition, different materials in this area had different effects on extrusion distortion. Machine head body was mainly used to transition the melt in the homogenization section to a narrow flow channel. According to the flow channel curve obtained by simulation, the front section of the conical flow channel was a hemispherical transition section. At the same time, a straight section with a certain length can ensure that the material had a certain extrusion pressure. The plasticized melt, which was pushed forward and rotated in the barrel by the screw, passed through the perforated plate and became linear motion. In addition, due to the blocking of the perforated plate, the resistance of the molten material to advance was increased, and the quality of the plasticization of the material in the barrel was improved. The core of this design is the detachable metal part. The replacement of this part can explore the effect of different external mechanical means on extrusion distortion, so as to obtain products with better hydrophobic properties.

Step 3. The screw temperatures of zones 1 to 3 of the single-screw extruder were set to 150 °C, 160 °C, and 165 °C. The die temperature was set to 165 °C. After the temperature was reached, we kept it warm for another hour. Then the blended pellets were put into the loss-in-weight hopper of the single-screw extruder to ensure uniform extrusion.

Step 4. Starting from a low speed, the screw was manually moved, and the speed was slowly increased. Sampling was performed at 20 r/min, 40 r/min, 60 r/min, and 80 r/min speeds. Sampling was performed several times in sections, and the samples were cooled and shaped in water.

Step 5. The additives were AC3, AC5, AC15, and 180. The LLDPE/additive ratio was 9:1, and the die temperature was set to 165 °C. Samples were taken several times at 20~80 r/min, and the samples were also cooled and shaped in water.

Step 6. The temperature of the third zone of the screw and the die temperature varied from 155 °C to 165 °C. There were four kinds of additives mentioned above. The samples were taken several times, and they were cooled and shaped in water.

### 2.3. Characterization

The microscopic morphology of the surface of the extrudate was observed by an optical microscope (DM2000, LEICA, Wetzlar, Germany) and a field emission scanning electron microscope (TM4000, HITACHI, Ibaraki, Japan). The contact angle of the water droplet of the prepared sample was passed through a droplet shape analyzer (SL200KB, KINO, Shanghai, China).

## 3. Result and Discussion

Different parameters, especially kinds of additive, die temperature and screw speed, have a significant impact on the surface morphology and hydrophobic properties of the product. In this section, their effects will be systematically investigated and discussed to find the best processing parameters. In addition, the relationship between surface morphology and hydrophobic properties will be discussed.

### 3.1. The Influence of Different Additives on Surface Morphology

**Figure 4:**
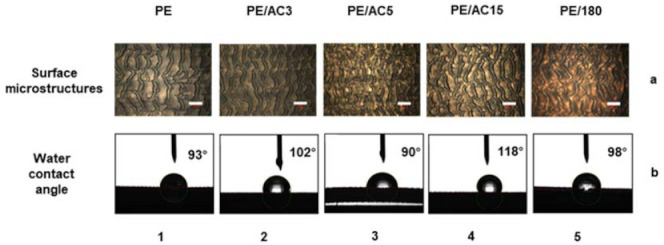
(**a**) The morphology and water contact angle of samples prepared with different additives in (**b**) micrometer scale. (**a**) The scale bar in the illustration was 400 μm. (1–5) respectively showed no additives, AC3, AC5, AC15, 180.

As shown in Figure 4, this shows the effect of additives on the surface morphology and hydrophobic properties of the prepared samples. In this figure (Figure 4a,b) is represented the microscopic characterization results and the water contact angle, while 1–5 represent the changes in the case of different additives. Therefore, Figure 4(a1) shows the water contact angle of the pure LLDPE sample. Similarly, the image named Figure 4b2 shows the microscopic morphology of LLDPE/AC3 at a blend ratio of 9:1. The rest of the pictures can be named and explained in the same way. The ratio of LLDPE/additives is 9:1. The die temperature and screw speed of all the prepared samples in Figure 4 were 165 °C and 80 r/min, respectively. According to the measurement results, the contact angle of the pure LLDPE material was 93°, while the contact angle of the samples prepared with additives had been enlarged to varying degrees (as shown in Figure 4b).

Steps for measuring water drop contact angle: Step 1. Establish a report. The sessile drop method was selected as the test type, and ADSA Young–Laplace equation was selected for automatic data processing. Step 2. Set parameters. In the Young–Laplace parameter, real drop was 90%, the threshold was automatically settled, and the function of removing blind side and noise was selected. Step 3. Adjust the area box to start the measurement. Step 4. Conduct manual fitting for each curve with poor fitting and export the data.

Based on the Wenzel–Cassie model, the increase in surface roughness is beneficial to enhance the hydrophobicity of the prepared samples [43]. The higher surface roughness can increase the gas phase ratio in the solid–liquid–gas interface. When water drops fall, higher surface roughness will retain more bubbles on the polymer surface. Due to the influence of surface tension, the wetting of the area under the bubbles is very difficult. In addition, the dense air bubbles in the layered structure will form an air cushion between the water droplets and the polymer surface. This ultimately results in a durable polymer hydrophobic surface.

From Figure 4 we can observe that the surface of the pure LLDPE was wavy, and cracks appeared in the respective areas of the wavy. The uniformity of the product was relatively poor. The size of the microstructures was large, the height was short, and the grooves between the microstructures were wide. In the area shown in Figure 4(a1), the number of microstructures was small, the surface of the structure was smooth, and there was almost no micro–nano composite structure. The roughness factor was low, the phase area fraction was large, and the water contact angle of the structure was 93°. From the results, we can see that LLDPE, as a densely entangled polymer, was prone to melt fracture during the extrusion process. Even at a higher extrusion temperature, when the rotation speed reached a certain range, the extrusion distortion was obvious. At the same time, it can be seen from the contact angle results that LLDPE had certain hydrophobic properties and was very suitable for the preparation of hydrophobic materials.

After AC3 was added to LLDPE, the ratio of LLDPE/AC3 was 9:1, and other conditions remained unchanged, but the surface microstructure had changed greatly. The microstructure was still wavy, but the size of the microstructure was significantly smaller than that of the pure material. There were fewer cracks, and the overall uniformity was relatively good. In the area shown in Figure 4(a2), there were more microstructures and the surface was relatively rough. The water contact angle was 102°, which was higher than that of pure LLDPE. This showed that the addition of the slip agent AC3 made the polymer more prone to extrusion distortion during the extrusion process. The changes in the size of the microstructure and the surface energy of the material improved the hydrophobic properties of the material surface.

When AC5 was selected as an additive and the screw speed was 80 r/min, the surface microstructure of the material was in an incomplete state. Although the size of the microstructure was small, it can be clearly seen that the uniformity of the distortion was poor. As shown in Figure 4(a3), the number of complete microstructures was small, and there were more places where the structure was broken as a whole. The contact angle of the prepared material surface was 90°, which was lower than that of pure LLDPE. However, when AC5 was used as an additive, the size of the microstructure was obviously smaller than other additives. In the case of lowering the temperature and increasing the speed, the hydrophobic properties of the microstructure may improve.

When the additive was AC15, as shown in Figure 4(a4), the size of the microstructure was smaller, the microstructure was blocky, and there were more complete microstructures in the area. The height of the microstructure was higher, and the size of the microstructure groove was smaller. The structural roughness factor was higher, the phase area fraction was lower, and the water contact angle was 118° when compared to other groups. The experimental results of this group also proved that the microstructure with a large roughness factor and a smaller phase area fraction had a larger contact angle.

When 180 was selected as the additive, as shown in Figure 4(a5), the shape of the microstructure was blocked. The height of the microstructure was higher, but the uniformity was poor. The number of complete microstructures in the area was relatively small. A higher microstructure corresponded to a larger roughness factor, but when the roughness exceeded a certain value, the contact angle decreased. The larger groove width of the structure means that the phase area fraction was larger, and the contact angle was also reduced.

In addition to the above qualitative analysis of the prepared samples, we selected different positions of the prepared samples to measure the surface microstructure size, microstructure shape, and uniformity of the microstructure of the product. After averaging, we came up with Table 1: The microstructure characteristics of the measured samples from different groups.

We compared the structural characteristic parameters of the five groups of samples from various aspects. Judging from the shape of the microstructure, pure LLDPE and LLDPE/AC3 (9:1) had a wavy microstructure at 165 °C and 80 r/min, and the contact angle of the sample was relatively small. However, LLDPE/AC15 (9:1) and LLDPE/180 (9:1) presented discontinuous lumps at 165 °C and 80 r/min. The contact angle of the sample was relatively large, and the hydrophobic property was better. In terms of the integrity and uniformity of microstructure size, the samples prepared by LLDPE/AC5 (9:1) had the worst integrity, the smallest contact angle and poor hydrophobic property. Compared with the number of microstructures of five groups of samples in a certain range, LLDPE/AC15 (9:1) had more and more uniform microstructures than other groups, and a larger contact angle. On the whole, LLDPE/AC15 (9:1) had a better performance in terms of hydrophobic properties. In subsequent experiments, AC15 was used as an additive to blend and extrude with LLDPE. At the same time, although the structural integrity of the samples prepared by AC5 was poor, the microstructure size was smaller, and so the subsequent experiments can be observed carefully. In the research, we also used high density polyethylene (HDPE) as material to explore the effects of additives on the hydrophobic properties of the products. The influence law was also in line with the results obtained in this paper.

### 3.2. The Influence of Die Temperature on Surface Morphology

**Figure 5:**
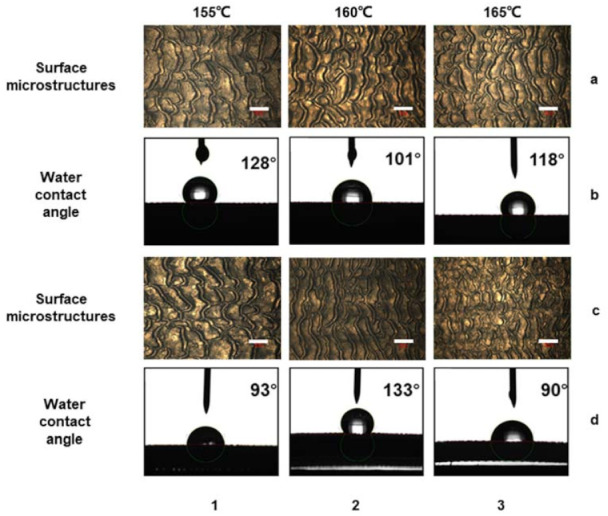
(**a**) When LLDPE/AC15 (9:1) was used as the material and the screw speed was 80 r/min, the changes in the morphology and water contact angle of the prepared sample with temperature on the micrometer scale (**b**). (**c**) When LLDPE/AC5 (9:1) was used as the material and the screw speed was 80 r/min, the changes in the morphology and water contact angle of the prepared sample with temperature on the micrometer scale (**d**). The scale bar in the illustration (**a**,**c**) was 400 μm. (1–3) showed the die temperature was 155 °C, 160 °C, 165 °C respectively.

After confirming the choice of AC15 as an additive, the influence of die temperature on surface morphology and hydrophobic properties was further studied. The additive ratio of all prepared samples shown in Figure 5a,b was LLDPE/AC15 (9:1), and the screw speed was 80 r/min. When the die temperature was 165 °C, the surface of the product presented a block-like microstructure with a size of nearly 160 μm. The height of the microstructure was higher and the groove was wider. There were many microstructures in the area, but the uniformity was poor. This means that the roughness factor was higher, but the phase area fraction was smaller. The contact angle reached 118°, and the hydrophobic property was better. When the temperature was lowered to 160 °C, the size of the microstructure on the surface of the product increased, and some of the structures appeared continuous and developed in a wave shape. The height of the microstructure became lower, the groove size became wider, and the uniformity of the structure became worse. At this time, the contact angle was reduced to 101°, and the hydrophobic property became worse. When the temperature dropped to 155 °C, the size of the surface of the product was basically stable, and there was no trend of further expansion. At this time, the size of the microstructure was larger and the height of the microstructure was lower, but a tiny structure appeared on the block structure. This combination of microstructure and nanostructure gave the block structure a rough texture, and the contact angle increased instead.

In addition, the experiment also observed the change of the microstructure morphology and contact angle with the temperature of the extruder head when AC5 was used as an additive. As shown in Figure 5c,d, the ratio of LLDPE to AC5 was 9:1, and the screw speed was 80 r/min. When the temperature was 165°, the microstructure on the surface of the product was incomplete. The contact angle of water droplets corresponding to the structure was 90°. As the temperature decreased, when the die temperature was 160 °C, the shape of the microstructure was already very complete and the uniformity was very good. The size of the microstructure was smaller and there were few cracks. The surface of the microstructure had a smaller rough structure, the contact angle was as high as 133°, and it had optimal hydrophobic properties. When the temperature was further reduced to 155°, the size of the microstructure became larger and the contact angle was 93°. It can be seen that the size of the microstructure had a great influence on the hydrophobic properties of the structure.

The experimental results show that lowering the temperature within a given range increases the size of the microstructure and decreases the product’s hydrophobic characteristics. When the temperature falls below a specific point, however, nanostructures develop on the surface of the microstructures, enhancing the hydrophobic property even further.

Nosonovsky [44] studied the stability of the superhydrophobic surface composite interface caused by different roughness surfaces. Bhushan [45] produced a hydrophobic surface with hierarchical roughness through micropattern replication and self-assembly of hydrophobic alkanes. This proved that hierarchical roughness was beneficial to hydrophobicity. Wu [46] used hot embossing technology to prepare a biomimetic hierarchical roughness hydrophobic surface, and also explored that the hierarchical roughness surface had better hydrophobic properties. This conclusion was further confirmed in this experiment. When AC15 was used as an additive to prepare samples, the optimal temperature for preparing hydrophobic surfaces with hierarchical roughness should be around 155 °C. When AC5 was used as an additive to prepare samples, the optimal temperature should be 160 °C. This also proved that temperature had different effects on different materials during the extrusion process.

### 3.3. The Influence of the Screw Speed on the Surface Morphology

**Figure 6:**
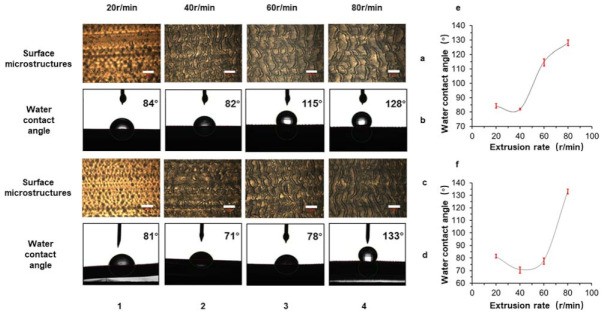
(**a**) When LLDPE/AC15 (9:1) was used as the material and the die temperature was 155 °C, the changes in the morphology and water contact angle of the prepared sample with screw speed on the micrometer scale (**b**). (**c**) When LLDPE/AC5 (9:1) was used as the material and the die temperature was 160 °C, the changes in the morphology and water contact angle of the prepared sample with screw speed on the micrometer scale (**d**). (**e**) The relationship between the screw speed and hydrophobic properties of the samples prepared in the case of LLDPE/AC15 (9:1). (**f**) The relationship between the screw speed and hydrophobic properties of the samples prepared in the case of LLDPE/AC5 (9:1). The scale bar in the illustration of (**a**,**c**) was 400 μm. (1–4) respectively showed that the screw speed was 20 r/min, 40 r/min, 60 r/min, 80 r/min.

A similar research process had also been applied to find the optimal screw speed. Figure 5 showed the results of the water contact angle and morphology of the prepared samples. Among them, the additives selected in Figure 6a,b,e were LLDPE/AC15 (9:1), the extruder head temperature was 155 °C, and the screw speed varied from 20 to 80 r/min. The additives selected in Figure 6c,d,f were LLDPE/AC5 (9:1), the extruder head temperature was 160 °C, and the screw speed varied from 20 to 80 r/min.

For the group of LLDPE/AC15, when the screw speed was 20 r/min, the surface of the sample had almost no microstructure, the sample was relatively smooth, and the contact angle was low. When the screw speed was increased to 40 r/min, the surface had certain microstructures, but the uniformity of the structure was poor and the contact angle decreased. With the increase of the screw speed to 60 r/min, the surface microstructure was gradually complete and the size was smaller. However, there were partial cracks and the groove was wider, so the contact angle was improved. The contact angle reached its maximum value at 80 r/min. The size of the microstructure at this time was complete. Although the size of the microstructure increased, the structure had layered roughness and exhibited a better hydrophobic property.

When the additive was AC5, the change in trend was similar to the previous group. At 40 r/min, the contact angle of the product surface reached the minimum, and at 80 r/min, the contact angle of the product was the largest. From this we can also see that as the temperature changed, the contact angle increased first and then decreased. The integrity of the structure had a greater impact on the hydrophobic property of the structure. The size of the microstructure and the layered roughness structure had a great influence on the hydrophobicity.

**Figure 7:**
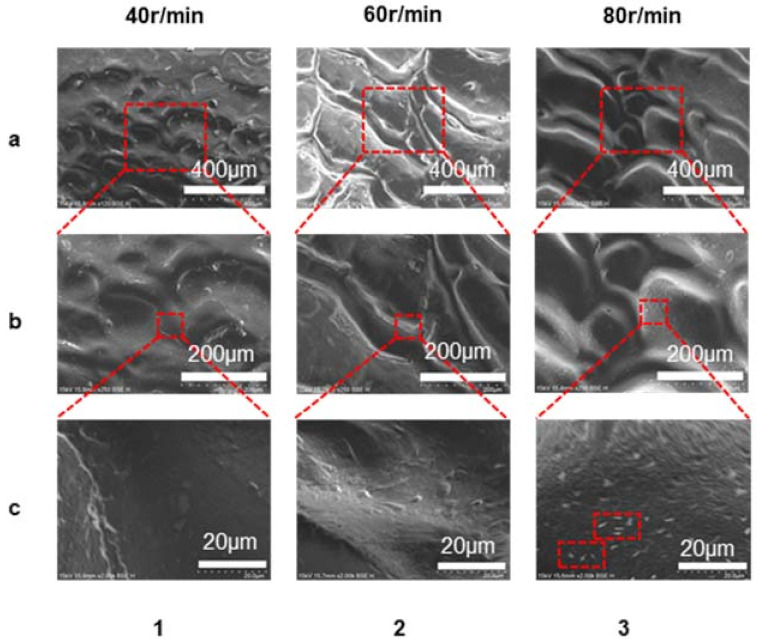
The morphology of LLDPE/AC15 (9:1) at different micron scales with screw speed when the die temperature was 155 °C. (**a**–**c**) showed the magnifications of 120, 250, and 2000 respectively. (1–3) respectively showed that the screw speed was 40 r/min, 60 r/min, 80 r/min.

We also used a scanning electron microscope to examine the microstructure on surface, as shown in Figure 7. We can see that the microstructure’s shape became more visible as the screw speed increased, which meant the microstructure height increased gradually with increasing screw speed. The magnifications of Figure 7a,b were 120 and 250, respectively. Figure 7c had a larger magnification of 2000. More details on the surface of microstructures can be observed this time, as shown in Figure 7(c3). The surface of this hierarchical roughness structure would lead to better hydrophobic property and a larger water contacting angle. Through the morphological change in the surface microstructures, we can see that with the increase of the screw speed, the size of the microstructures continued to increase, and at the same time nanostructures began to appear on the surface of the microstructures. Even at 80 r/min, although the microstructure size was large, the product still had better hydrophobic properties due to the hierarchical roughness structure.

Hirvi [47] obtained the following conclusions through MD simulation of the contact angle on the rough surface of polyethylene. When the wall surface roughness factor was the same, the contact angles of the nano-water droplets on the nano-fence-shaped (wave-shaped) or nano-square column matrix (block-shaped) rough wall surface were not much different. With the increase in the roughness factor, the contact angle began to increase, and then basically did not change. As the phase area fraction increases, the contact angle basically remained unchanged at first, and then gradually decreased. The experimental results of each group also proved this conclusion very well.

In general, if you want to obtain products with better hydrophobic properties, you can choose AC15 as an additive; the die temperature is set to 155 °C, and the screw speed is 80 r/min. AC5 can also be used as an additive, the die temperature is set to 160 °C, and the screw speed is 80 r/min. Both methods can obtain samples with larger water contact angles.

## 4. Conclusions

In this article, using LLDPE as the material, we explored a new method for preparing the hydrophobic surfaces of polymers through the phenomenon of melt fracture during the extrusion process. Through parameter optimization, it was determined that the optimal process parameters of the LLDPE sample prepared by extrusion is 160 °C and 80 r/min, and the optimal additive is AC5. The water contact angle can reach 133° under the above optimal parameters. It is worth mentioning that the entire time period of the extrusion process is continuous and is suitable for continuous sheet forming. By changing the kinds of polymer, extruder die size, screw speed, and special treatment of the extrusion runner, the hydrophobic properties of the product are expected to be further improved. Therefore, the method proposed in this paper will be a simple and economical method for large-scale industrial production of polymer hydrophobic surfaces.

## Figures and Tables

**Table 1:** Surface characteristics of measured samples from different groups.

Material	Microstructure Width (μm)	Microstructure Shape	Number of Microstructures in the Area (1 mm × 1.2 mm)	Water Contact Angle (°)
LLDPE	243 ± 23	Wavy	4	93 ± 3
LLDPE/AC3 (9:1)	208 ± 19	Wavy	5	102 ± 1
LLDPE/AC5 (9:1)	150 ± 20	Lumpy	6	90 ± 2
LLDPE/AC15 (9:1)	163 ± 17	Lumpy	10	118 ± 4
LLDPE/180 (9:1)	214 ± 24	Lumpy	5	98 ± 1

## Data Availability

The data isn’t disclosed, and the author can be contacted if necessary.
